# Biological effects of magnetic fields emitted by graphene devices, on induced oxidative stress in human cultured cells

**DOI:** 10.3389/fbioe.2024.1427411

**Published:** 2024-07-11

**Authors:** Sara Franceschelli, Pierdomenico D’Andrea, Lorenza Speranza, Federica De Cecco, Teresa Paolucci, Valeria Panella, Alfredo Grilli, Stefano Benedetti

**Affiliations:** ^1^ Department of Medicine and Aging Sciences, University “G. d’Annunzio” Chieti- Pescara, Chieti, Italy; ^2^ Uda-TechLab, Research Center, University “G. d’Annunzio” of Chieti-Pescara, Chieti, Italy; ^3^ School of Medicine, University “G. d’Annunzio” Chieti-Pescara, Chieti, Italy; ^4^ Department of Innovative Technologies in Medicine and Dentistry, University “G. d’Annunzio” Chieti- Pescara, Chieti, Italy; ^5^ Department of Medical Oral Sciences and Biotechnology (DiSmob), Physical Medicine and Rehabilitation Unit, G. D’Annunzio University of Chieti-Pescara, Chieti, Italy

**Keywords:** oxidative stress, antioxidant enzymes, electromagnetic fields, quantum dots, reactive nitrogen species

## Abstract

Many recent studies have explored the healing properties of the extremely low-frequency electromagnetic field (ELF-EMF) to utilize electromagnetism for medical purposes. The non-invasiveness of electromagnetic induction makes it valuable for supportive therapy in various degenerative pathologies with increased oxidative stress. To date, no harmful effects have been reported or documented. We designed a small, wearable device which does not require a power source. The device consists of a substrate made of polyethylene terephthalate and an amalgam containing primarily graphene nanocrystals, also known as quantum dots. This device can transmit electromagnetic signals, which could induce biological effects. This study aims to verify the preliminary effects of the electromagnetic emission of the device on leukemic cells in culture. For this purpose, we studied the best-known effects of magnetic fields on biological models, such as cell viability, and the modulations on the main protagonists of cellular oxidative stress.

## Highlights

The device was made of graphene nanocrystals (quantum dots):

• does not affect the viability in Jurkat cells
• alleviates the oxidative stress in H_2_O_2_-activated cultures stimulating the activity of ROS-neutralizing antioxidant enzymes such as SOD and CAT.
• prevents the accumulation of H_2_O_2_, restoring the activity of GR and GPX.
• can represent a non-pharmacological inhibitor of NO.

## 1 Introduction

Many studies have demonstrated the effectiveness of electromagnetic stimulation at Extremely Low Frequencies (ELF) (Markov and Colbert, 2000). Electromagnetic stimulation is induction to all intents and purposes: a time-varying electromagnetic field (EMF) generates an electric current in a conductor according to electrology (Maxwell’s rules). This also applies to biological tissue exposed to an EMF. The biological response is the change in transmembrane potential caused by the induced microcurrents (Pall, 2013). The specific targets of the inducing fields are mainly the cell membrane, namely, the Cl^−^, Na^+^ and K^+^ channels, which are responsible for creating the endogenous tissue electromagnetic field. Scientific works show how exogenous EMFs can increase membrane permeability to Potassium, Calcium, Magnesium and Lithium (Liboff and McLeod, 1988; De Fabritiis et al., 2008). The effect on ion channels is due to the principle of electro-conformation of alpha-helices, i.e., the structural, temporary, electro-mediated modification of the alpha-helix conformation of trans-membrane proteins (Tsong et al., 1989). In another study on ion pumps, the effect of exogenous technological fields on molecular dynamics is highlighted on Na/K and Cl pumps (Ikehara et al., 1998). It is possible to generate micro-currents in biological targets using all electromagnetic spectrum bands (Romanenko S et al., 2017). Exogenous EMFs also work on forced water flow and proton flow. Since 1992, it has been known that protein channels regulate the flow of water molecules activated by cellular energy. These proteins are called aquaporins; they are located within the cell membrane’s lipid bilayer and allow water to flow bi-directionally Madeira A, Moura TF, Soveral G. Detecting Aquaporin Function and Regulation. Front Chem. 2016 Feb 1; 4:3. doi: 10.3389/fchem. 2016.00003. PMID: 26870725; PMCID: PMC4734071. Exogenous EMFs act on these proteins using electro-conformational coupling, i.e., an alternating field or current causes the electrically induced conformation of the protein molecule to oscillate between two states, synchronized with the field or applied current. Aquaporins and ion channels are particularly sensitive to such perturbations (Brown and Sivak, 2020) The action of induced currents occurs on the hydrogen bonds of the C = O and N-H groups, temporarily changing the molecular structure. In the case of aquaporins, the opening and closing of the pores are synchronous to the frequency of the fields induced by the exogenous stimulation with the associated passage of water. Very weak EMFs represent a medium which is particularly effective in therapy. The non-invasiveness of this non-ionizing and athermic radiation and the documented absence of side effects or harmful effects to date places electromagnetic induction in the sphere of supportive therapies, making it possible to obtain appreciable results in various degenerative-type pathologies sustained by increased oxidative stress, chronic tissue inflammation and uncontrolled apoptotic phenomena (Tofani, 2022).

Recent studies show that the concomitance of multiple molecular damage to cellular substrates causes many diseases. The cause of such damage is mainly related to reactive oxygen species (ROS) (Jomova K et al., 2023). The altered production of ROS produces an increased production of oxidized proteins, glycosylation end-products and lipid peroxidation. This condition, denominated oxidative stress (OS), also forms toxic species, i.e., peroxides, alcohols, aldehydes, ketones and oxidative modifications to nuclear and mitochondrial DNA. ROS are primarily produced in the mitochondria during aerobic metabolism. They play a vital role in inter- and intracellular signalling, as well as in defence against microorganisms. However, ROS can also be generated by external factors like UV radiation and pollution or by changes in the immune system caused by inflammatory reactions. The stated condition can increase the risk of developing chronic diseases. The organism has a built-in system of antioxidants that can combat the harmful effects of ROS. Enzymatic substances like superoxide dismutase (SOD) and catalase (CAT), along with non-enzymatic molecules like glutathione and vitamins (such as A, C, and E), work together to detoxify the cell from reactive oxygen species (Jena et al., 2023). In addition to ROS, reactive nitrogen-containing molecules, including free radical nitric oxide (•NO), play a role in immune cells and many other cell types. Three isoforms of nitric oxide synthase (NOS) produce nitric oxide (NO). These are endothelial (eNOS), neuronal (nNOS), and inducible (iNOS) (Patruno A et al., 2010). While eNOS and nNOS are enzymes regulated by calcium/calmodulin, iNOS is a cytokine-induced form. It leads to the robust synthesis of NO in immune cells and other cell types. iNOS plays a role in immune actions. NO is not cytotoxic per se at the physiological level. However, it can react spontaneously with superoxide at high concentrations to form highly reactive peroxynitrite, damaging the DNA and proteins. Additionally, to the Fenton chemistry pathways, the peroxynitrite pathway poses a major oxidative stress-related threat to biological molecules. In physical therapy, electromagnetic induction can be carried out using various instruments capable of radiating electromagnetic waves in a systemic mode (mats containing solenoids, radial field concentrator emitters for local therapies). Such equipment is usually used in healthcare facilities or at the patient’s home and requires a connection to the mains or at least a power supply (Jeon et al., 2015). This small, wearable device, which does not require a power source, consists of a polyethylene terephthalate substrate on which an amalgam containing mainly graphene nanocrystals (quantum dots) is layered. Because of ambient light, this device induces a delocalization of electrons and simultaneously an emission of photons by the Quantum Dots (QDs) present. A semiconductor nanocrystal is characterized by its bandgap energy, which is required to excite an electron and move it from one electronic band to another at a higher energy level. This excitation pattern creates an “electron-lacuna” pair known as the “exciton”. Upon its “return” to its ground state, the exciton emits energy as a photon. When the size of the semiconducting nanocrystals is in the range of 2–10 nm, like Bohr’s exciton beam, they gain specific electric and optical properties. This mechanism leads to transmitting electromagnetic signals that can induce biological effects. The best documented and understood property is the inverse relationship between nanocrystal size and energy bandgap; in other words, as nanocrystal size decreases, the energy band gap increases, and the corresponding excitation/emission wavelengths decrease. This is called the “Quantum Effect,” which compares the excitation and emission profiles of QDs with different sizes (Abraham and Weiss, 2004; Sobhanan et al., 2023). This study examines the immediate effects of electromagnetic emissions from a device on cultured leukemic cells. We investigated how magnetic fields, which are known to affect biological models, impact cell viability and oxidative stress. The experimental setup involved creating a precise electromagnetic field exposure system and exposing leukemic cell cultures to specific ELF-EMF. This research is significant because ELF-EMF has been shown to influence biological models, potentially altering cell growth, differentiation, and apoptosis. Understanding their effects on leukemic cells could provide valuable insights into their potential implications for human health. Additionally, the findings of this study could potentially support traditional therapies against oxidative-based pathologies in the future by identifying new therapeutic targets or enhancing the efficacy of existing treatments. The broader implications of this research might extend to developing guidelines for safe exposure levels to electromagnetic fields, informing public health policies, and advancing biomedical engineering applications aimed at improving patient outcomes.

## 2 Materials and methods

### 2.1 Device

**FIGURE 1:**
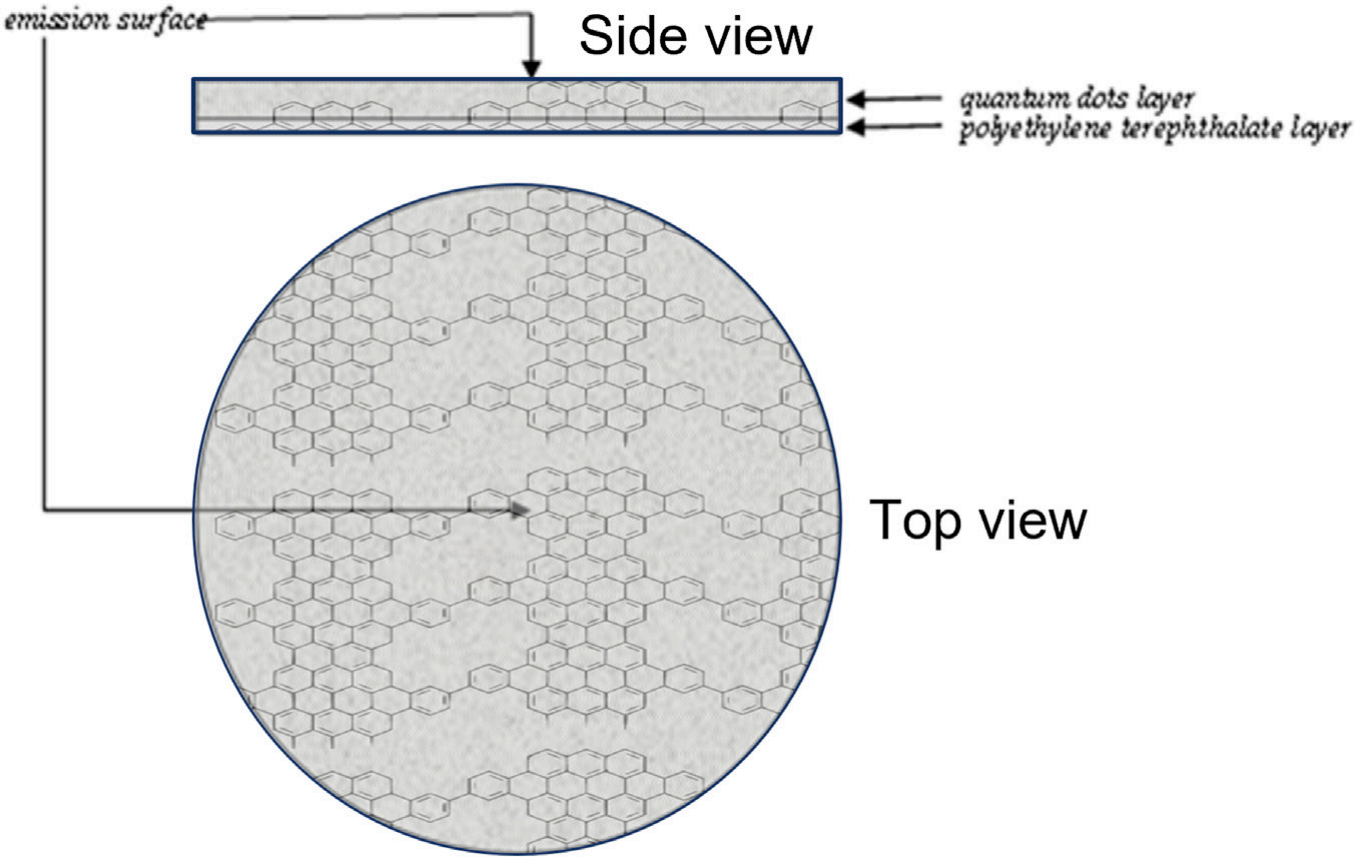
Device Assembly. The Device comprises a Polyethylene Terephthalate (Mylar^®^) support on which Quantum Dots graphene nanocrystals are stratified. Due to the effect of the electron-hole pair triggered by sunlight, it emits energy in the form of photons at 800 nm.

The Device was designed as a circular polyethylene terephthalate (Mylar^®^) substrate (6 mm, 4 mm) on which an amalgam containing mainly graphene nanocrystals (quantum dots) is layered; due to sunlight, the electro-hole pair that is created determines an emission peak at 800 nm (Figure 1). The Device displays the excitation wavelength (λex = 485 nm) and the emission wavelength (λem = 525 nm), Full Width at Half Maximum (FWHM: 70 nm, 800 nm) and Intensity less than 0.1 mW.

### 2.2 Cell cultures

Jurkat T cells. a type of human T-cell leukaemia lymphoblastoid cells (DSMZ ACC 282) were grown and maintained in RPMI-1640 supplemented with 10% fetal calf serum, L-glutamine (4 mM), penicillin (100  U mL−1), and streptomycin (100  U mL−1). Cells growing exponentially were treated at 2,106 cells/mL density. The cells were obtained from the Deutsche Sammlung von Mikroorganismen und Zellkulturen (DSMZ, Braunschweig, Germany). To induce oxidative stress, H_2_O_2_ (Sigma) was added to the Jurkat culture at concentrations of 100 μM. In the control cells, water was added to the samples instead of H_2_O_2_ to obtain unstressed samples. The device was inserted on the Petri culture dish as the H_2_O_2_ was added. After a 24 h incubation period, we assessed cellular responses to oxidative stress by measuring the cell viability, generation of free radicals, and conducting Western blot analysis as described below. In a preliminary experiment, we utilized a spectrometer (PCE-CRM 40-PCE ITALY s. r.l) to precisely control the emission wavelength of the device. The exposure duration of 24 h was found to effectively counteract oxidative stress while keeping the emission values constant.

### 2.3 Cytotoxicity assay

The Methylthiazolyldiphenyl-tetrazolium bromide (MTT) assay (Sigma-Aldrich, St. Louis, MO, United States) assessed the metabolic activity of Jurkat cells treated with the device. Cells were seeded in duplicate 5 × 10^5^ in 100 µL of complete medium in a 96-well plate. Briefly, cells were maintained in a fresh, serum-free medium for 4 h to synchronize their growth. After removing the medium, a fresh serum-free medium was added, and the cells were incubated with the device for 24 and 48 h. The MTT reagent was added to each well with a 0.5 mg/mL concentration. After the incubation at 37°C for 2 h, the yellowish solution was converted to intracellular dark blue water insoluble MTT formazan crystals by mitochondrial dehydrogenases of living cells. The MTT/medium was removed and replaced with an equal volume of DMSO (Sigma-Aldrich, St. Louis, MO, United States) to solubilize cells and the intracellular formazan crystals. The spectrophotometer (SpectraMax^®^ 190, Molecular Devices, CA,United States) was used to measure the absorbance at 570 nm of the resulting purple solution.

### 2.4 ROS detection

ROS production by Jurkat cells exposed to the device was performed by an NBT (nitroblue tetrazolium) assay, as previously described (Franceschelli et al., 2023). Enzyme activity (superoxide anion O_2_^−^ production) was reported as an NBT reduction stimulation index (SI). The SI was calculated by taking treated and control cells’ optical density (OD) ratio. The SI value for the control was 1.

### 2.5 Nitric oxide synthase (NOS) activity

The oxyhemoglobin assay was conducted to detect nitric oxide production from NOS, as detailed in the previous report (Tiboni et al., 2021). The reaction mixture for assessing NOS activity consisted of CaCl_2_ (1.6 mM), l-arginine (10 µM), calmodulin (11.6 mg/mL), tetrahydrobiopterin (6.5 µM), dihydronicotinamide-adenine dinucleotide phosphate (NADPH, 100 µM), and oxyhemoglobin (3 mM) in 4-(2-hydroxyethyl)-1-piperazineethanesulfonic acid (HEPES, 100 mM) at pH 7.5, in a final volume of 1 mL. iNOS activity was evaluated under calcium-free conditions. The detection of methemoglobin, the product of the reaction between nitric oxide and oxyhemoglobin, was performed at 576 nm (e = 12.000 M^−1^ cm^−1^).

### 2.6 Glutathione assay, glutathione peroxidase (GPX) and glutathione reductase (GR) activity

The level of reduced glutathione (GSH) in cells was measured using the method outlined by Franceschelli et al. (2018). To do this, Jurkat cells were cultured in 6-well plates and treated as indicated. The cells were lysed using RIPA lysis reagent on ice for 30 min. After lysis, the cell lysate was clarified by centrifugation at 100,00× g for 10 min at 4°C, and the supernatant was collected for further analysis. T-GSH was measured using the 5,5-dithio-bis (2-nitrobenzoic) acid (DTNB)-GSSG reductase recycling method. The amount of GSSG was determined by measuring the 5-thio-2-nitrobenzoic acid (TNB) produced by the reaction of reduced GSH with DTNB. The auto-microplate reader was used to measure the rate of TNB formation, which was recorded at 412 nm. To determine the concentration of reduced GSH in the sample, the GSSG value was subtracted from T-GSH. The colour was read at 412 nm, and the GSH values are expressed as nmol GSH per mg of proteins.

GPX activity was measured spectrophotometrically by recording the decrease in NADPH oxidation at 340 nm using a previously described method (Franceschelli et al., 2016). At 25°C, the activity of one unit of enzyme was defined as the oxidation of 1 µmol of NADPH per minute.

The GR activity was determined using spectrophotometry (SpectraMax^®^ 190, Molecular Devices, CA, United States) at 340 nm and 25°C. One unit of enzyme activity is the oxidation of 1 mmol of NADPH per minute at 25°C.

### 2.7 Western blot analysis

Western blot analysis was carried out using the following antibodies: rabbit monoclonal catalase (sc-50508, Santa Cruz Biotecnology), rabbit polyclonal SOD 2 (sc- 30080, Santa Cruz Biotecnology), and mouse monoclonal β-actin (Santa Cruz Biotechnology, Inc., Dallas, TX, United States). The blots were incubated for 1 h at room temperature using goat anti-mouse secondary antibody (Sc-2005; 1:2000; Santa Cruz Biotechnology) or polyclonal goat anti-rabbit secondary antibody (Sc-66931; 1:5000; Santa Cruz Biotechnology). The nitrocellulose was scanned using a computerized densitometric system (Bio-Rad Gel Doc 1000, Milan, Italy). To adjust for variability in protein loading, protein levels were normalized to the housekeeping proteins β-actin and expressed as a percentage of the vehicle control.

### 2.8 RNA extraction, reverse transcription, and real-time PCR

RNA was isolated from cells using 1 mL QIAzol lysis reagent (Qiagen, Hilden, Germany) per manufacturer’s protocol. The total RNA concentration was measured using a NanoDrop 2,000 UV–Vis spectrophotometer (Thermo Scientific, Waltham, MA, United States). To transcribe 1 µg of total RNA, a QuantiTec Reverse Transcription Kit with integrated genomic DNA contamination removal (Qiagen, Hilden, Germany) was used, following the manufacturer’s instructions. cDNA was employed for real-time PCR assays and performed in triplicate using GoTaq qPCR Master Mix (Promega, Madison, WI, United States), as previously described (Pesce et al., 2018). The experiment was carried out using the following conditions: 2 min incubation at 95°C, followed by 40 cycles consisting of 30 s at 95°C, 1 min at 60°C, and 30 s at 68°C. Specific human primer pairs, CAT, SOD2 and iNOS were used to evaluate the expression of target molecules. The CAT primer pair had a Forward Primer sequence of 5′-CAT​TCG​ATC​TCA​CCA​AGG​TTT​GGC​C-3′ and a Reverse Primer sequence of 5′-AGC​AGG​TAG​GGA​CAG​TTC​ACA​GG-3’. The SOD2 primer pair had a Forward Primer sequence of 5′- CTG​CTG​GGG​ATT​GAT​GTG​TGG-3′ and Reverse Primer sequence of 5′-TGC​AAG​CCA​TGT​ATC​TTT​CAG​T-3. The iNOS primer pair had a Forward primer sequence of 5′-CAT​TGC​TGT​GTC​CAT​AGT​TTC-3′ and Reverse Primer sequence of 5′-CAG​GAC​CTA​AGT​TCA​GCA​TCT​C-3’. Using the ΔCt method, the relative expression of each gene was normalized to the 18s gene, where ΔCt = Ct(CAT,SOD2)− Ct18s. Relative fold changes in gene expression were determined by the 2^−ΔΔCT^ method, where ΔΔCt = ΔCtexperimental sample − ΔCtcontrol sample.

### 2.9 Statistical analysis

Quantitative variables are summarized as the mean value and standard deviations (SD). To assess the accuracy of fold change data, the 95% confidence interval (95% CI) and standard error (SE) were determined. A two way ANOVA analysis non parametric test followed by Tukey’s multiple comparisons test was applied to evaluate the significance of differences. All tests were two-tailed. The threshold of statistical significance was set at *p* = 0.05. Data analysis was performed on GraphPad Prism 8 Software, version 8.4.

## 3 Results

### 3.1 The device does not affect the viability of and mitigates oxidative stress in Jurkat cells

**FIGURE 2:**
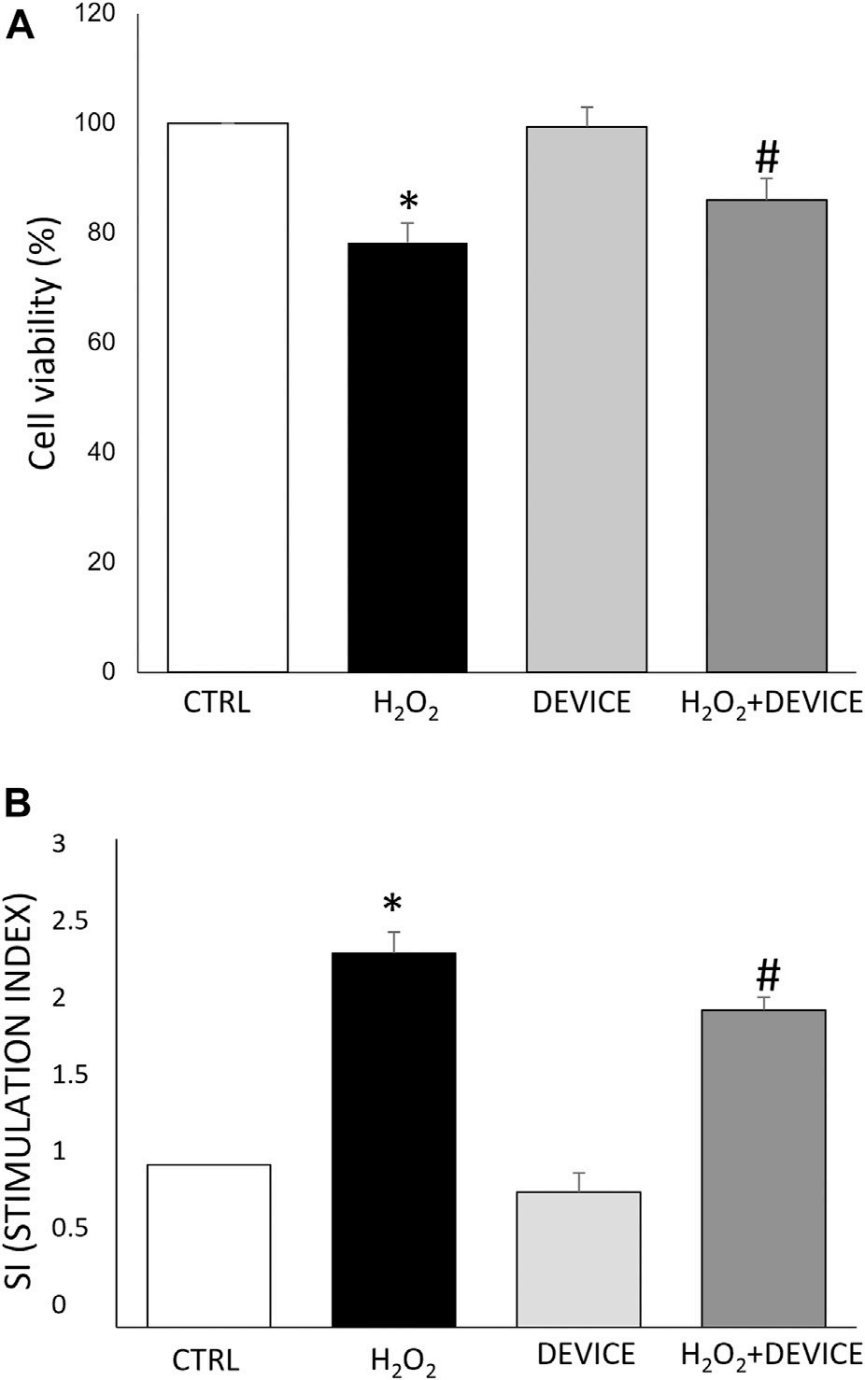
Effect of the device on Jurkat cells. **(A)** Cell viability analysis. Cells were exposed to the device for 24 h. Cell viability was measured by MTT assay as reported in Materials and Methods. Data are reported as % of viability to control cells (n = 3). Cell viability value of 100% was assigned to control cells. **(B)** The antioxidant activity of the device against oxidative stress H_2_O_2_-induced was measured by an NBT test. Results were registered as stimulation index (SI). SI value of 1 was assigned to control cells. Each bar represents mean ± SEM (n = 3). *p < 0.005 vs. CTRL; #*p* < 0.05 H_2_O_2_.

Preliminary experiments were carried out to determine the optimal concentration of H_2_O_2_ and the duration of exposure. A concentration of 100 µM resulted in a solid pro-oxidant reaction without compromising the viability of the cells, and 24 h of exposure to the device was able to counteract oxidative stress (data not shown). The MTT assay (24 h) was used to assess the cytotoxicity impacts of the device on Jurkat cells treated or not with H_2_O_2_. The device did not affect cell viability (see Figure 2A). Furthermore, the superoxide anion (O_2_^−^) radical-scavenging activity was also measured using a non-enzymatic method. The generation of O_2_^−^ was markedly inhibited (∼50%) after exposure to the device with respect to cells H_2_O_2_ stimulated (Figure 2B). As a result, the device was used in subsequent experiments to test its antioxidant activity further.

### 3.2 Effect of the device on the cellular antioxidant network

**FIGURE 3:**
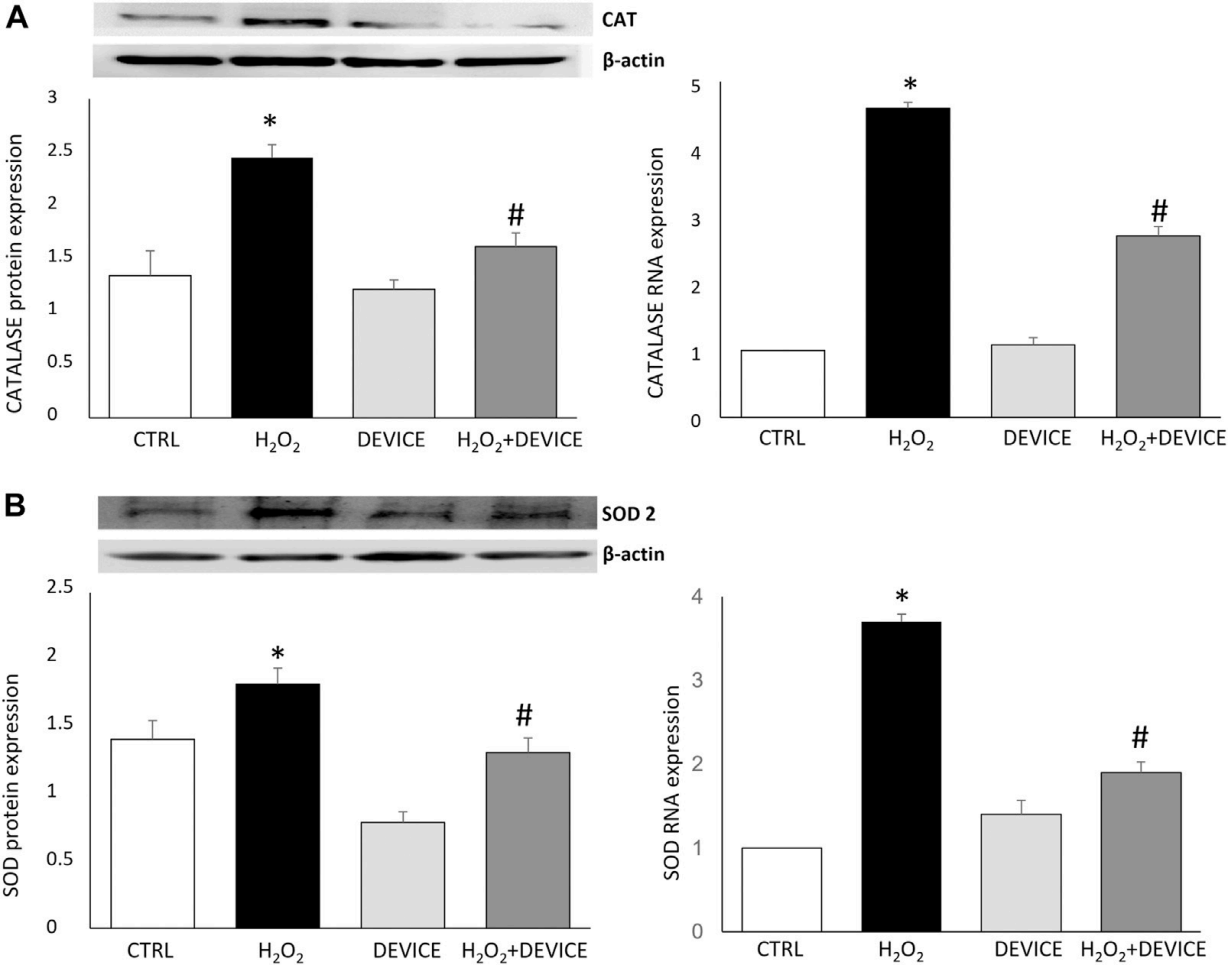
Effect of the device on antioxidant enzymes. Western blot analysis (left) and Real-Time PCR (right) showing the intensities of Catalase **(A)** and SOD-2 **(B)** in Jurkat cells. Values were normalized against the β-actin. The data shown are mean ± SEM (n = 3). *p < 0.05 vs. control. #*p* < 0.05 vs. H_2_O_2_ treated cells.

Next, to test the antioxidant capacity of the device, we evaluated by measuring its effect on the activity of SOD and CAT, which are the main antioxidant enzymes, in cells exposed to H_2_O_2_. When comparing the activated cells with the device, it was found that the device significantly increased the activity of CAT (*p* < 0.001) and SOD (*p* < 0.001). This indicates that the device has antioxidant capacity (as shown in Figure 3). Therefore, the device can reduce ROS levels and relieve the oxidative stress in H_2_O_2_-challenged Jurkat cultures by stimulating the activity of ROS-neutralizing antioxidant enzymes such as SOD and CAT.

### 3.3 Effect of the device on GSH-Related enzymatic activities

**FIGURE 4:**
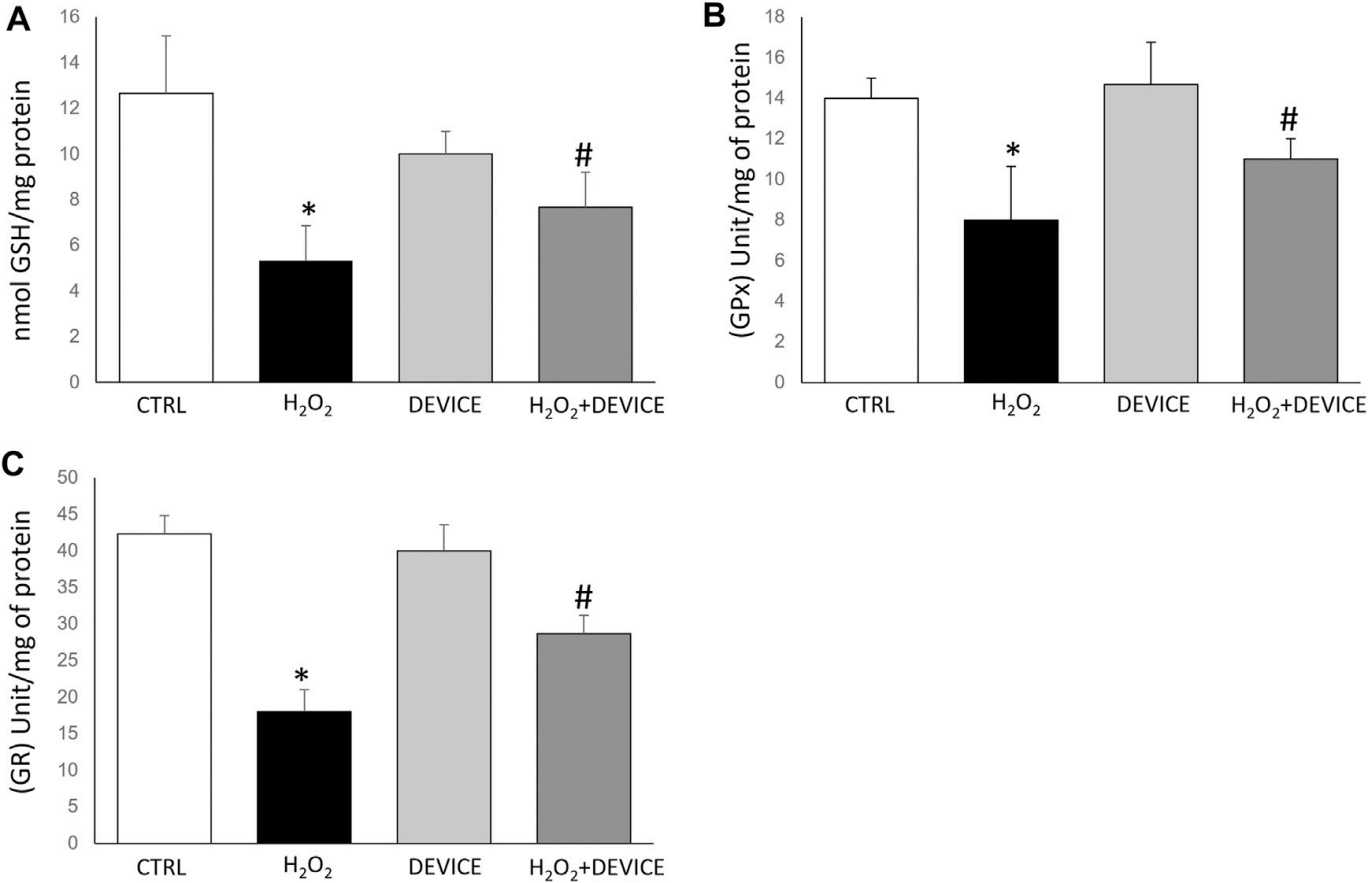
Effect of device exposure on glutathione-redox status. Cells were lysed, and the intracellular concentration of glutathione (GSH) **(A)** was quantified. Glutathione peroxidase **(B)** and Glutathione reductase **(C)** activity was normalized based on the total protein content of cell lysates. One unit of enzyme activity was defined as 1 µmol of NADPH oxidized/min. Data are expressed as mean ± SD of three independent experiments performed in triplicate. *p < 0.05 device-exposed vs. control cells. GPx: glutathione peroxidase; GR: glutathione reductase.

Glutathione peroxidase (GPx) and the GSH system are essential for maintaining cellular homeostasis. In addition, we examined the effect of device exposure on intracellular glutathione-redox balance, which plays a critical role in regulating the antioxidant response. Upon H_2_O_2_ treatment, Jurkat cells showed GSH depletion, confirming the pro-oxidant state (Figure 4A). Intracellular antioxidant enzyme activities involved in the GSH–redox homeostasis (glutathione peroxidase (GPx) and glutathione reductase (GR)) were assessed. In H_2_O_2_-treated Jurkat cells, a reduction in GPx activity was observed. However, when the cells were exposed to the device, the GPx activity increased after exposure, with respect to H_2_O_2_-activated cells (Figure 4B). A similar trend was observed for GR activity (Figure 4C). The device prevents the accumulation of H_2_O_2_ by restoring the activity of glutathione reductase and glutathione peroxidase. In cells exposed to the device, GPx effectively removes peroxides. It converts the reduced GSH into oxidized glutathione disulfide (GSSG), which is then converted back into the antioxidant GSH by GR.

### 3.4 Effect of the device on the iNOS/NO system

**FIGURE 5:**
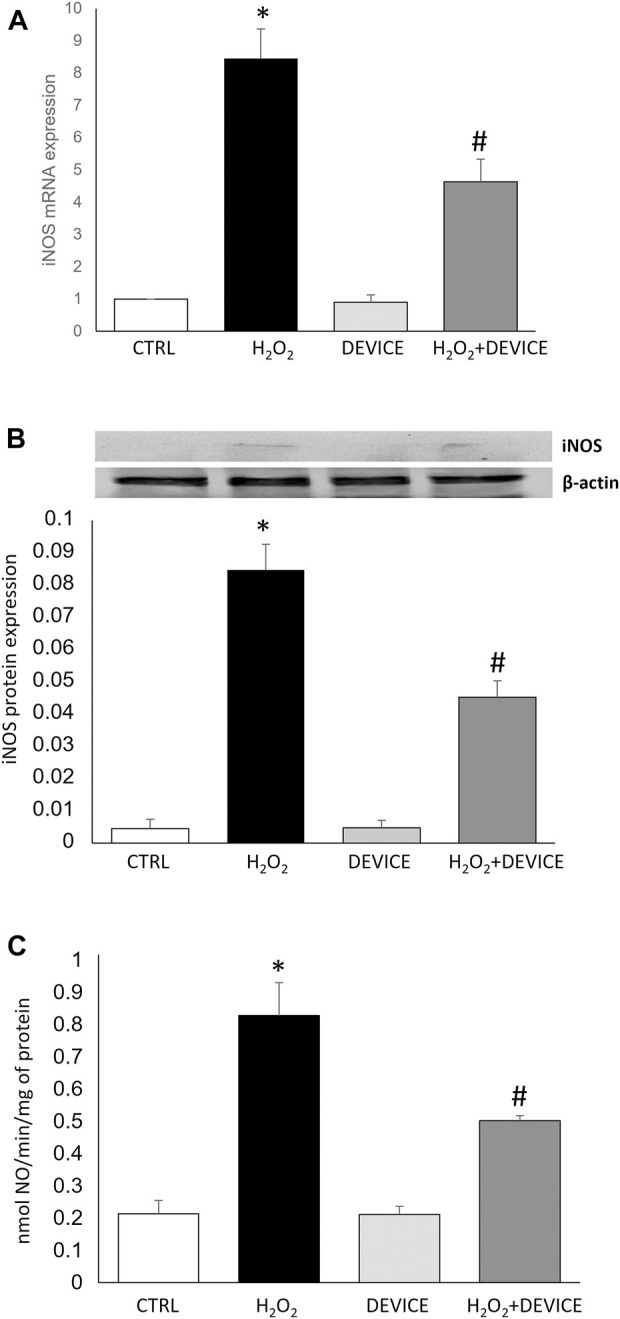
Effect of the device on iNOS/NO system. **(A)** mRNA expression of iNOS expressed in Jurkat Cells, measured by quantitative real time PCR and normalized to GADPH. Bars represent median values for sample category. The results of three independent experiments are expressed as mean ± SD. **(B)** Western blots are illustrated for iNOS protein extracted from Jurkat cells. A representative experiment of a separate experiment (n = 3) was shown. The gels were counterstained for β-actin to ensure the application of equivalent cell extracts. **(C)** iNOS enzymatic activities in Jurkat cells exposed or not to H_2_O_2_ or Device. Each histogram represents the mean ± SD of data from three independent experiments. NOS activity is expressed in nmol NO/min/mg prot. *p < 0.05 H_2_O_2_ vs. control cells; #*p* < 0.05 H_2_O_2_ + Device vs. H_2_O_2_ cells.

In the next set of experiments, we determined the molecular effect of the device on iNOS/NO signalling. Nitric oxide (NO) is continuously generated in cellular systems and is an essential physiological mediator. On the other hand, oxidative stress is involved in signal transduction pathways to induce nitric oxide synthase (iNOS). The induction of iNOS appears when cells are subjected to various stresses, such as exposure to H_2_O_2_. NO acts in both directions for cell protection and damage, and its excessive production causes cellular alterations (Pesce et al., 2018). Our data confirm that a significant increase in iNOS expression is observed in induced oxidative stress. Exposure to the device negatively affects the upregulation of this inducible enzyme, with a consequent reduction in NO levels (Figure 5).

## 4 Discussion

Bioelectric phenomena play a vital role in life processes. All living organisms have intrinsic characteristics, including the ability to separate, transport, and store electrical charge (Santini et al., 2018). One essential requirement is the transfer of electrons in electrochemical communication between molecules. An EMF is associated with the mobile and charged electron. Electromagnetic forces are responsible for the structure of matter from atoms to more complex substances and play a pivotal role in cell division and other processes in eukaryotic cells (Cichoń et al., 2017). Electron transfer is probably the most widespread and essential process in chemical transformation. Examples are receptor chemistry and the cell signalling mechanism (Gökçek-Saraç et al., 2023). The electro-chemistry plays an essential role in biological functioning. In a recent hypothesis, electrostatic force is a factor in receptor-ligand action based on the molecular electrostatic potential associated with ions and dipoles. The cell can couple adenosine triphosphate (ATP) hydrolysis energy directly to endergonic processes by transferring a phosphate from ATP to another molecule, which becomes more reactive. Different studies on the electrochemical effects of metal cations mainly involve Mg, Zn, Fe, Ca, and Cu. Of these, calcium has received the most attention concerning cell communication and receptor binding (Kovacic et al., 2008). Calcium and other alkaline earth cations modify the potential adjacent to negatively charged bilayer membranes. Electromagnetic phenomena are associated with charged radicals and moving electrons. Recent research has reported that gap junctions are involved in cell-cell signalling. In addition to the primary effects of electromagnetic fields on ionic charges and dipolar matter, cellular biochemical reactions and channel transport processes depend on the nature of the field (Schofield et al., 2020). The interaction of signalling systems with low-energy electromagnetic fields can produce metabolic responses in the body (Luben, 1991). The applied field may modify existing signal transduction processes in cell membranes, thus producing both transduction and biochemical amplification of existing field effects. A practical example includes the physiological effects of the external field in healing bone fractures (Cadossi et al., 2020). There is a good correlation between dipole moments and receptor activity. Investigators have acquired information on physiological events in which weak forces of up to 0.5 pN play a regulatory role in, for example, ion channel function (Goychuk, 2018). Biological studies *in vitro* have focused on the nature of the signal transduction pathway involved in responding to electromagnetic fields. It is likely that electromagnetic fields directly interact with electrons in DNA to stimulate biosynthesis through electron transport/charge flow in DNA. Studies on electromagnetic field induction of stress response proteins suggest the potential application of electromagnetic fields in two biomedical areas: cytoprotection and gene therapy.

Since the early 1970s, electromagnetic fields have been used therapeutically to accelerate the healing of bone fractures and soft tissue wounds. Epidemiological studies have suggested that EMFs can also induce adverse health effects and that biological systems perceive EMFs as a possible danger. However, exposure to EMFs also has beneficial effects; as the interpretation of the mechanisms grows, the specific requirements for field energies are better defined, and the range of treatable diseases is expanding (Kıvrak et al., 2017). These include nerve regeneration, wound healing, graft behavior, diabetes and myocardial and cerebral ischemia, among other conditions (Wang and Zhao, 2010). The modulation of cell surface chemical events by electromagnetic fields indicates that increased amplification of initial weak effects is associated with binding hormones, antibodies, and neurotransmitters to their specific sites. Calcium ions play a crucial role in this amplification (Saliev et al., 2014). These studies provide new concepts of cell-to-cell communication. In the cell aggregates that form the tissues of larger animals, cells are separated by narrow fluid channels that become particularly important in cell-to-cell signalling. These channels operate as windows to the electrochemical world surrounding each cell. Hormones, antibodies, neurotransmitters, and chemical cancer promoters move along them to reach binding sites on cell membrane receptors. These narrow fluid ‘gutters’, usually no wider than 150 A, are also preferred pathways for intrinsic and environmental electromagnetic fields, as they offer much lower electrical impedance than cell membranes. Although this intercellular space (ICS) constitutes only about 10 per cent of the conducting cross-section of a typical tissue, it carries at least 90 per cent of any imposed or intrinsic current, directing it along the cell membrane surfaces (Funk RH et al., 2009). Several stranded protein molecules protrude inside the cell into this narrow ICS. Their glycoprotein tips form the glycocalyx, which detects chemical and electrical signals in the surrounding fluid. Their negatively charged tips form receptor sites for hormones, antibodies, neurotransmitters, and many metabolic agents, including cancer promoters. These charged terminals constitute an anatomical substrate for the initial detection of weak electrochemical oscillations in the interstitial fluid, including field potentials arising from adjacent cell activity or as tissue components of environmental fields. Many living organisms, including humans, use EM energy to regulate critical cellular systems. Ligand-receptor effects may occur due to field influences on intracellular signalling molecules or membrane transport substances. Enzyme cascades or ion fluxes can amplify such effects. Non-linear mechanisms can amplify weak MF effects at extremely low frequencies. A recently published review focuses on the results described in the literature on the response of cells and tissues to electromagnetic fields (Sun et al., 2022). Ring-shaped electric fields found in nature are essential for cell surface interactions and crucial for the normal development of the organism and its physiological functions: Based EMFs, e.g., in wounds and bone fractures. Another study suggests that applied ELF-EMF reduces oxidative stress induced by global cerebral ischemia and decreases possible adverse effects that free radicals might have on the brain. Studies in cell biology have shown that weak electromagnetic fields can influence various critical cellular functions. EMF targets in cells are signal transduction cascades. Furthermore, EMFs influence specific gene transcription, cell growth, and membrane-mediated signal transduction processes, particularly regarding the Ca^2+^ transport system. Effects of EMF on mitochondrial functions, cell growth and transformation, neutrophil signal transduction, cell apoptosis, gene expression and lipid peroxidation of the biological membrane have been examined. It is well established that low-frequency EMF fields (<300 Hz) induce a range of biological effects, such as increased enzyme reaction rates and transcription levels for specific genes (Goodman and Blank, 2002). Antioxidative enzymes such as SOD, CAT, and GPx deactivate or scavenge free radicals before they attack cellular components. SOD scavenges the superoxide anions and generates hydrogen peroxide, and CAT and GPX convert hydrogen peroxide to water and O_2_ (Pesce et al., 2018). Results obtained from our experiments showed that antioxidant enzymes were reduced with the H_2_O_2_ stimulation compared to control, but the levels improved markedly after EMF treatment. The induction of the expression of the antioxidant enzyme by exposure to electromagnetic fields provides information on how these fields interact with cells and tissues. The results of these and other studies have also provided essential clues on the interaction of electromagnetic fields with cellular systems at the molecular level. Detailed information on the interaction of electromagnetic fields with charged movement and their influence on enzyme reaction speed in cell-free systems can be found. The scientific literature provides evidence that EMFs influence cell physiology by altering redox-related processes, inducing an increase in ROS production associated with decreased ROS scavenging activity. However, ELF-EMFs can modulate oxidative stress (Santini et al., 2018). The coupling of EMFs with biological systems depends on the frequency range of the signals used and their characteristics, such as amplitude, modulation, waveform, and polarization. Using EMFs with specific characteristics is a non-invasive therapeutic method for various diseases. Recent evidence has indicated that exposure to EMFs can suppress the production of ROS, the overproduction of which can alter cellular elements by damaging antioxidant mechanisms. Short-term EMF exposure has been reported to activate systems that control oxidative balance in the rat brain, reduce ROS production and increase the production of the antioxidant enzyme MnSOD in neuroblastoma cells (Jeon et al., 2015; Patruno et al., 2015). Elevated levels of ROS can stimulate the activation of transcription factors, such as NF-κB, which can upregulate the expression of iNOS. ROS can directly influence iNOS gene expression by modulating signaling pathways involved in iNOS regulation (Franceschelli S et al., 2016). The relationship between increased ROS and iNOS underscores the critical importance of maintaining redox balance in cells to prevent excessive nitric oxide production and oxidative stress. Strategies aimed at reducing ROS levels or inhibiting iNOS activity hold potential for mitigating the detrimental effects of this interplay, offering promising therapeutic avenues for conditions characterized by oxidative stress and inflammation (Nediani and Dinu, 2022). Our research findings have demonstrated that utilizing our innovative device significantly decreases the expression and activity of iNOS, a key contributor to inflammation and oxidative stress when overexpressed. This reduction in iNOS activity underscores the device’s effectiveness in regulating nitric oxide levels, presenting promising implications for managing conditions characterized by inflammation and oxidative stress. Our device offers non-pharmacological solutions for cellular protection and balance maintenance, representing a significant advancement in the field of medical engineering. By leveraging electromagnetic emissions, the device shows promise in enhancing the cellular antioxidant system, inhibiting nitric oxide, and counteracting the harmful effects of reactive oxygen species. Further research is imperative to fully understand the device’s potential therapeutic applications and to engineer therapeutic systems based on electromagnetic fields. Future studies could explore clinical applications in specific conditions such as neurodegenerative diseases, cardiovascular disorders, and autoimmune conditions. Additionally refining the device’s design could enhance its efficacy and broaden its clinical utility. This engineering-driven advancement holds promise for developing novel treatment options and improving outcomes across a spectrum of health conditions. It signifies a significant stride forward in the pursuit of innovative, non-invasive, and effective medical interventions rooted in engineering principles.

## Data Availability

The original contributions presented in the study are included in the article/Supplementary Material, further inquiries can be directed to the corresponding author.
